# Repellency of Essential Oils and Plant-Derived Compounds Against *Aedes aegypti* Mosquitoes

**DOI:** 10.3390/insects16010051

**Published:** 2025-01-07

**Authors:** April D. Lopez, Sophie Whyms, Hailey A. Luker, Claudia J. Galvan, F. Omar Holguin, Immo A. Hansen

**Affiliations:** 1 Department of Biology, New Mexico State University, Las Cruces, NM 88003, USA; aprlopez@nmsu.edu (A.D.L.); hailey13@nmsu.edu (H.A.L.); 2 NatPro Centre, School of Pharmacy and Pharmaceutical Sciences, Trinity College Dublin, 2 Dublin, Ireland; whymsso@tcd.ie; 3 Department of Plant and Environmental Sciences, New Mexico State University, Las Cruces, NM 88003, USA; cjgalvan@nmsu.edu (C.J.G.); frholgui@nmsu.edu (F.O.H.)

**Keywords:** repellent, essential oils, concentrations, mixtures, arm-in-cage, clove, cinnamon, eugenol, geraniol, 2-PEP

## Abstract

Plant-based oils have a long history of use as insect repellents. In an earlier study, we showed that in a 10% concentration, geraniol, 2-phenylethl propionate, and the plant-based essential oils clove and cinnamon effectively protected from mosquito bites for over 60 min. To expand on this study, we reanalyzed our GC–MS data to identify the short organic constituents of these oils. We then used an arm-in-cage assay to test the repellency of different concentrations and combinations of these oils and pure compounds. We found a sigmoidal relationship between the complete protection time from mosquito bites and the concentration of these oils. The complete protection times we recorded for combinations of these oils suggest an absence of additive effects. The results of this study can inform the development of novel, effective, and plant-based insect repellents.

## Simple Summary

Plant-derived essential oils are a complex mixture of various compounds. They have been used as an alternative to synthetic mosquito repellents since ancient times. Currently, there are limited and at times contradictory scientific data on their efficacy. The aim of this study was to explore the mosquito repellency of two essential oils—clove and cinnamon—and four pure plant-derived oil compounds—2-PEP, geraniol, eugenol, and eugenyl acetate. We tested these oils at concentrations up to 10% and in different combinations, with the exception of eugenyl acetate that was only tested at a 10% concentration. We found a sigmoidal relationship between concentration and protection from mosquito bites for all these oils, except for eugenyl acetate. We found that concentrations of more than 5% often did not result in longer protection times than 5% concentrations. Mixing two effective repellents did not provide longer protection times than those of individual oils. Our findings are an important contribution to our understanding of plant-derived mosquito repellents and stress the importance of the scientific efficacy testing of plant-derived mosquito repellent products.

## 1. Introduction

Mosquitoes transmit major vector-borne and re-emerging infectious diseases around the world [1,2]. Specifically, the yellow fever mosquito, *Aedes aegypti* (Linnaeus, 1762), is a vector of diseases such as yellow fever, dengue, chikungunya, and Zika [3,4,5,6]. *Ae. aegypti* is distributed across all continents, except Antarctica, and is heavily present in tropical and subtropical zones [7]. Due to their rapid geographical expansion, the global risk for mosquito-borne diseases continues to increase despite advancements in mosquito population control measures [8,9].

Among control measures, the proper use of personal insect repellents can decrease the risk of contracting mosquito-borne diseases [10,11]. Effective repellents available commercially include synthetically derived active ingredients such as DEET, picaridin, and IR3535 [12]. Although these synthetic repellents are highly effective, there is growing consumer interest in alternatives that are plant-derived and considered more “natural” [13,14,15].

Mosquito repellents interact with chemoreceptors and alter mosquito host-seeking behaviors [16]. These receptors are located in their antennae, maxillary palps, labella, and tarsi [17,18]. Different classes of receptors located in these organs are critical for detecting human volatile compounds [19,20]. When a mosquito approaches a host, these chemoreceptors are used to determine host suitability [21]. The exact mode of action of many active ingredients in both synthetic and natural repellents is unknown [22].

In 1987, the United States Environmental Protection Agency (EPA) released a list of active ingredients eligible for minimum-risk pesticide status, which includes several plant-based oils [23]. The EPA 25(b) list of active ingredients eligible for minimum-risk pesticide products is a published document containing ingredients that are exempt from the FIFRA (Federal Insecticide, Fungicide, and Rodenticide) act.

There is abundant literature on the use of essential oils as mosquito repellents. Cinnamon oil, clove oil, and geraniol have been tested as mosquito repellents by many different groups [24,25,26,27]. Using a common homemade extraction protocol for three different clove extracts, Tan et al., 2019 used a surface, landing, and feeding assay to study repellency against *Culex quinquefasciatus* (Say, 1823) and *Ae. aegypti* [27]. Their study found eugenol, ß-caryophyllene, and eugenyl acetate to be the major constituents of the clove oil extracts. Using the equivalent of a 6–7% concentration for arm-in-cage assays, they found significantly lower repellency against both *Cx*. *quinquefasciatus* and *Ae. aegypti* compared with a 1% DEET control. Using the same clove oil formulations in an arm-in-cage assay, Miot and coworkers found similar results [28]. Several studies have investigated the repellency and insecticidal effects of cinnamon oil and its major constituents: eugenol and cinnamaldehyde [29,30]. Using a modified arm-in-cage assay, Uniyal et al. found 5% cinnamon oil from *Cinnamomum zeylancium* (Presl, 1823) to be an effective mosquito repellent for up to one hour [31]. The plant-derived compound “geraniol” has been shown to have significant repellency against mosquitoes in both spatial and contact repellency assays [32,33]. A study by Hao and coworkers demonstrated that *Aedes albopictus* (Skuse, 1894) mosquitoes, after a long exposure to geraniol, had altered host-seeking and blood-feeding behavior in an arm-in-cage assay [34]. A study conducted in 1999 by Barnard tested the repellency of different concentrations and combinations of essential oils from the EPA 25(b) list on *Aedes aegypti* and *Anopheles albimanus* (Wiedemann, 1820) mosquitoes [35]. A modified arm-in-cage assay was used to measure complete protection times (CPTs) against mosquito bites. The authors tested concentrations of essential oils from 5 to 100% using ethanol as carrier. Among all concentrations and combinations tested, the only mixture that repelled *Ae. aegypti* as well as a 25% DEET solution was a mixture of 75% clove oil and 25% thyme oil. In 2023, our group tested 21 active ingredients from the EPA 25(b) list on *Ae. aegypti* using an arm-in-cage assay [36]. This assay was used to measure the complete protection times (CPTs) from mosquito bites conferred by these active ingredients at 10% concentrations. We identified seven active ingredients that significantly protected from mosquito bites. Of these, four provided CPTs of over 60 min—cinnamon oil, clove oil, 2-PEP, and geraniol. A chemical analysis using GC–MS was performed to identify individual compounds within the tested active ingredients.

In the current study, we re-analyzed the GC–MS data from our previous study to investigate correlations between compound composition and CPT. We further expanded our investigation of cinnamon oil, clove oil, 2-PEP, and geraniol. First, we explored the impact of the concentration of these ingredients on CPT. We tested these ingredients in arm-in-cage assays at 1, 3, 4, 5, and 10% concentrations. We found that concentrations over 5% did not confer any significantly longer CPTs, except for 2-PEP. Next, we studied the effect of combining these top-performing active ingredients together in a 1:1 ratio at a 10% total concentration. We observed no significant additive effects. Lastly, we selected and further investigated two single compounds—eugenol and eugenyl acetate—found in high concentrations in cinnamon and clove oil. We measured the CPTs conferred by these two compounds at different concentrations. Our results suggest that eugenol is the major repellent compound in clove and cinnamon oil, while eugenyl acetate provided no significant protection from mosquito bites.

The findings from this study provide useful information for the future development and formulation of mosquito repellents derived from plant-based oils.

## 2. Materials and Methods

### 2.1. Mosquito Rearing

Two to three-week-old adult female *Ae. aegypti* mosquitoes from the strain UGAL (University of Georgia Laboratory) were used exclusively for all experiments. The UGAL strain was received from Alexander Raikhel’s laboratory at the University of California Riverside. Approximately 600 mosquito eggs were hatched in 32 × 42 × 6 cm^3^ pans filled with 2.5 L of DI water. The larvae were fed cat food pellets (Special Kitty, Walmart Stores Inc., Bentonville, AR, USA) ad libitum, and the water was changed as needed. The larvae were stored in an incubator set to 27.8 °C and 74% relative humidity. Pupae were transferred to a 200 mL dish filled with water that was stored in a 30 × 30 × 30 cm^3^ BugDorm-1 Insect-Rearing cage (Bug Dorm Company, Taichung, Taiwan). Adult mosquitoes were allowed to feed ad libitum on a 20% sucrose solution delivered in a 100 mL Erlenmeyer flask with a sucrose-solution-saturated cotton wick at the top. Sucrose solution flasks were replaced on a weekly basis. Adult mosquitos in rearing cages were stored in an insectary room set at a temperature of 27 °C, relative humidity of 80%, and light/dark cycle of 14/10 h, respectively. The mosquitoes used in this study were not blood-fed at any time.

### 2.2. Sample Preparation

Samples were prepared by diluting individual plant-based oils (Table 1) in an organic lotion base (chemistrystore.com, accessed on 8 August 2023). All samples were made to a total volume of 5 mL in 15 mL centrifuge tubes. The dilution series treatments were made *v*/*w*, plant-based oil to organic lotion base. The organic lotion base was measured in grams to provide the most accurate and consistent measurement. Individual plant-based oils were measured by volume and added to the organic lotion base. Each treatment was well mixed using a vortex for 60 s immediately before testing.

### 2.3. Re-Analysis of Gas Chromatography/Mass Spectrometry (GC–MS) Data

The GC–MS data reported in a previous study were re-analyzed. Kovats retention indices were calculated according to the method described by Luker et al. (2023) [36]. Compound identifications were putatively assigned by spectral matching to the GC/MS library using an 80% similarity cutoff and a retention index (RI) tolerance of 50. Following this, a quantitative analysis of identified compounds was performed by peak area normalization. The area of all identified peaks was assessed, and the quantity of each individual component in each oil was calculated and reported as percentage concentration. Every identified component was then sorted in terms of its compound class. The following classes were detected in our analysis: monoterpene hydrocarbon (MH), oxygenated monoterpene (OM), sesquiterpene hydrocarbon (SH), oxygenated sesquiterpene (OS), oxygenated diterpene (OD), ester (E), ketone (K), aldehyde (A), and other compound (OC). The re-analyzed GC–MS data can be found in Supplemental File S1.

### 2.4. Composition Analysis

**Figure 1:**
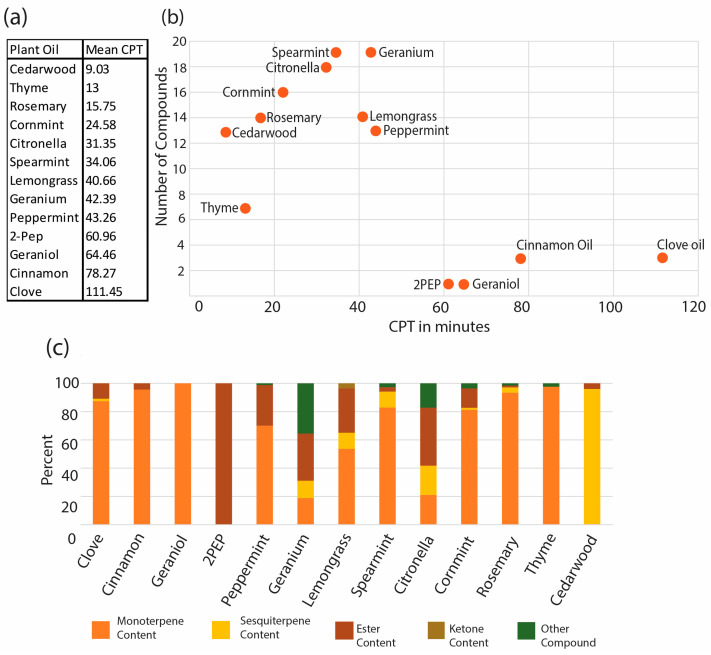
Plant-based oils with long CPTs are composed of few compounds/compound types. (**a**) Rank chart of CPTs of plant-based oils. Shown are plant-based oils ranked from lowest to highest based on mean complete protection time determined by Luker and coworkers [25]. Plant-based oils were ordered by CPT from highest to lowest. (**b**) Diagram of compound quantities plotted against CPT. Shown are the number of compounds identified in each plant-based oil with its corresponding CPT in minutes over laid with a polynomial trend line R^2^ = 0.54. (**c**) The relative distribution of compound types in plant-based oils. Shown are stacked bar graphs of percentages of different compound types identified in each oil.

Graphical representations of compound quantity and compound type were generated in Excel using the scatter plot function overlaid with an exponential trend line and the stacked bar plot charts, respectively (Figure 1b,c).

### 2.5. Arm-in-Cage Assay

The arm-in-cage assay was conducted following a modified version of the published EPA product performance guidelines for insect repellents [37,38]. Mosquitoes between 2 and 3 weeks old were sugar-starved for two to four hours prior to experimentation. Then, 25 female mosquitoes were transferred to a 30 × 30 × 30 cm^3^ Bug Dorm insect-rearing cage that was modified with a clear plexiglass side for observation. An ambient temperature of 22–27 °C and an approximate humidity of 40% was maintained throughout all experiments. The number of trials for each treatment was distributed evenly between male and female volunteers, with at least two males and two females per treatment. An elbow-length polyethylene glove (RoyalPaper.com, Royal, Dayton, OH, USA) was prepared by cutting out an 8.5 × 10 cm^2^ area in the forearm region of the glove. This cut-out region was the only part of the volunteer’s skin the mosquitoes had direct contact with during this assay. The volunteer used the scentless “Free & Clean” hand soap (seventh generation^®^) to wash their forearm and hand. The area was well-rinsed with water, then sanitized using 70% ethanol, and thoroughly dried with a clean paper towel. The volunteer then inserted their arm into the pre-cut glove. Two layers of cloth medical tape were used to adhere the edges of the cut-out area to the volunteer’s arm. Adequate biting pressure was established by inserting the volunteer’s gloved arm into a pre-prepared mosquito-infested Bug Dorm cage. Upon gloved-arm insertion into the cage, a stopwatch was started. If the volunteer received a mosquito bite within 60 s, the time was recorded as the control and the cage was used for treatment experiments. If the above requirement was not met, the cage was discarded. All bites were visually confirmed as proboscis insertion into the skin while all four mosquito tarsi had direct skin contact. The volunteer was instructed to immediately shake off a biting mosquito.

For experiments, 170 μL of a sample (2 μL/cm^2^) was applied to the volunteer’s exposed skin by reverse pipetting the emulsion with a 1 mL pipette with the tip cut off. The emulsion was spread evenly throughout the treatment area with the same pipette tip. As soon as the treatment area was fully coated, a stopwatch was started. The volunteer initially inserted their treated, gloved arm into the mosquito-filled cage for 15 min or until treatment failure, defined as the first mosquito bite. If a first bite did not occur during this time, the volunteer removed their arm from the cage and re-inserted it at the 30 min time mark for 5 min and then in regular 30 min intervals for 5 min at a time until treatment failure. Treatment failure was measured as complete protection time (CPT), defined as the entire time from treatment application up to the first bite in minutes. After the first bite, a second bite (confirmation bite) within 30 min of the first bite was necessary for the first bite to be valid. If a confirmation bite did not occur within 30 min after the first bite, the volunteer continued to reinsert their arm in the regular 30 min intervals for 5 min at a time or until another first bite was recorded and confirmed by a second bite in the same manner as stated above. We tested clove oil, cinnamon oil, geraniol, 2-phenylethyl propionate, and eugenol in 1, 3, 4, 5, and 10% concentrations. We only tested eugenyl acetate at a 10% concentration. We combined clove oil, cinnamon oil, geraniol, and 2-phenylethyl propionate in 1:1 binary mixtures, not to exceed 10% total EO concentration, and compared them to 5% and 10% single oil concentrations. All raw data can be found in Supplemental File S2.

### 2.6. Ethics Declaration

All experiments conducted in this study have been reviewed and approved by the New Mexico State University Institutional Review Board (IRB). We confirm that we followed all guidelines mentioned in our current application—(22010) “Insect and Tick-Repellent Research”, which expires 09/2029. The Environmental Protection Agency’s (EPA’s) protocol “OPPTS 810.3700. Insect repellents for human skin and outdoor premises.” was used as a guide to perform our arm-in-cage assay [39]. All volunteers were given and signed an informed consent form. Vulnerable persons (i.e., minors, pregnant and nursing women, prisoners, immune-compromised individuals, and people with severe reactions to mosquito bites) were excluded from this study [40]. Participants were advised to avoid alcohol, tobacco, and any scented products at least 12 h prior to this study. Four to five volunteers with a balanced ratio of males and females were recruited and performed the assay for each control and experimental group. Volunteers were between 21 and 54 years old. The recruitment of volunteers is addressed in our ethics declaration.

### 2.7. Statistical Analyses

Complete protection times (CPTs) in minutes were analyzed using GraphPad Prism10.0.0 [41]. For the dilution series, the data were entered using the XY table format. For all other analyses, a one-way ANOVA followed by Dunnett’s multiple comparisons test were used and followed by the compact letter display function [42,43].

## 3. Results

### 3.1. GC–MS Analysis of Plant-Based Oils

#### 3.1.1. Chemical Composition of Oils

We re-analyzed a GC–MS-derived dataset from our previous study on plant-based oils and individual compounds from the EPA 25(B) list [36]. We identified a total of 72 compounds across 13 different plant-based oils/compounds (Supplemental File S1).

We found that many of the analyzed oils had complex constituent profiles. Four of the oils had relatively few constituents identified. After a cut off threshold of 1% was applied to the relative percent concentration, three constituents were identified in clove oil—87.49% eugenol, 10.82% eugenyl acetate, and 1.68% aromadendrene. In cinnamon oil, we identified a total of three compounds—95.63% eugenol, 2.26% benzyl benzoate, and 2.12% eugenyl acetate. Geraniol, a monoterpene, and phenylethyl propionate (2-PEP), an ester, were the pure compounds used in this study.

#### 3.1.2. Repellency Profiling and Chemical Class Composition of Oils

Plant-based oils from the EPA 25(B) list were previously tested by Luker and collaborators at a 10% (*v*/*v*) concentration in an organic lotion base using the arm-in-cage assay [36]. DEET protected from mosquito bites for 6 h, at which point the experiment was ended. The mean CPTs ranged from 1.73 min for sesame oil to 111.45 min for clove oil (Figure 1a). Figure 1b shows the CPTs of individual oils plotted against the number of individual compounds we identified in these oils. The four oils with the highest CPTs, clove, cinnamon, geraniol, and 2-PEP, are composed of relatively few compounds. The compound classes per plant-based oil showed a similar trend (Figure 1a).

### 3.2. Repellency Efficacy of Plant-Based Oil Dilutions

**Figure 2:**
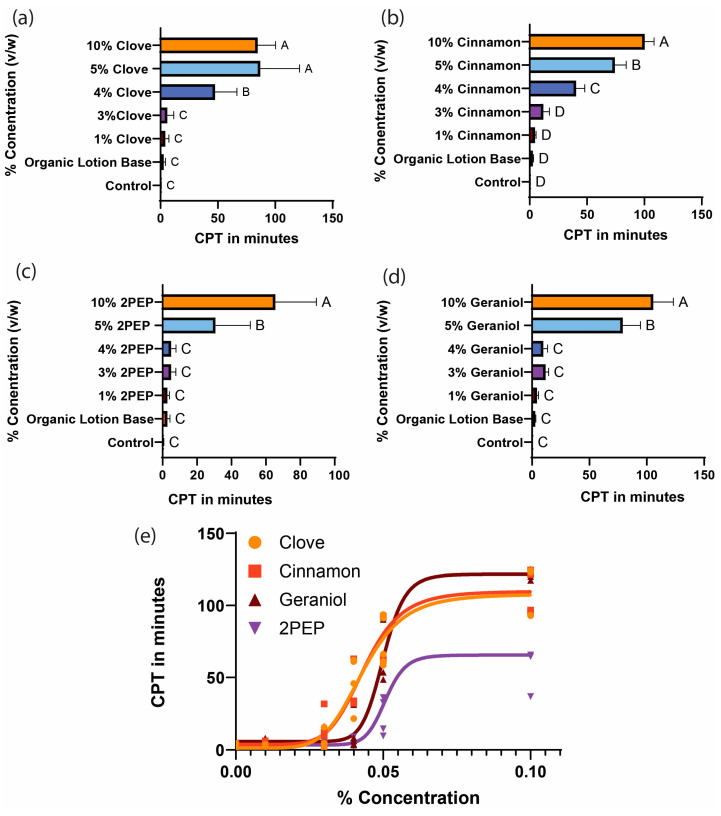
The relationship between plant-based oil concentration and CPT. (**a**–**e**) Complete protection times were measured using the arm-in-cage assay. (**a**–**d**) A one-way ANOVA followed by Dunnett’s multiple comparison test were used to calculate the statistical significance (*p* < 0.05) between the mean and standard deviation of each group. Experimental groups that share the same letter indicate no statistical difference, while groups that do not share the same letter indicate a statistically significant difference (*p* < 0.05) between the groups. (**a**) Clove oil dilution series. (**b**) Cinnamon oil dilution series. (**c**) 2PEP dilution series. (**d**) Geraniol dilution series. (**e**) Individual data points overlaid with a four-parameter sigmoidal curve model of the relationship between the concentration of plant-based oils to CPT. EC50 values are as follows: clove = 4.3%, cinnamon = 4.4%, geraniol = 5.0%, and 2PEP = 3.4%. The Hillslope values are as follows: clove = 6.2, cinnamon = 6.9, geraniol= 13.6, and 2PEP = 15.03. The goodness of fit was determined based on corresponding R^2^ values: clove R^2^ = 0.92, cinnamon R^2^ = 0.93, geraniol R^2^ = 0.96, and 2-phenylethyl propionate (2PEP) R^2^ = 0.80.

We chose two oils and two pure compounds to further test because they were previously shown to confer CPTs of over 60 min from mosquito bites. We measured the CPTs of these oils at lower concentrations up to 10% (Figure 2). A 0% concentration refers to the solvent, organic lotion base alone. A non-linear regression model was fitted to the dilution series data for each tested plant-based oil. Our model shows a low CPT prior to a steep increase around 4% concentrations and a plateau to 10% concentrations for all four plant-based oils tested.

### 3.3. Mosquito Repellent Efficacy of Different Mixtures of Oils

**Figure 3:**
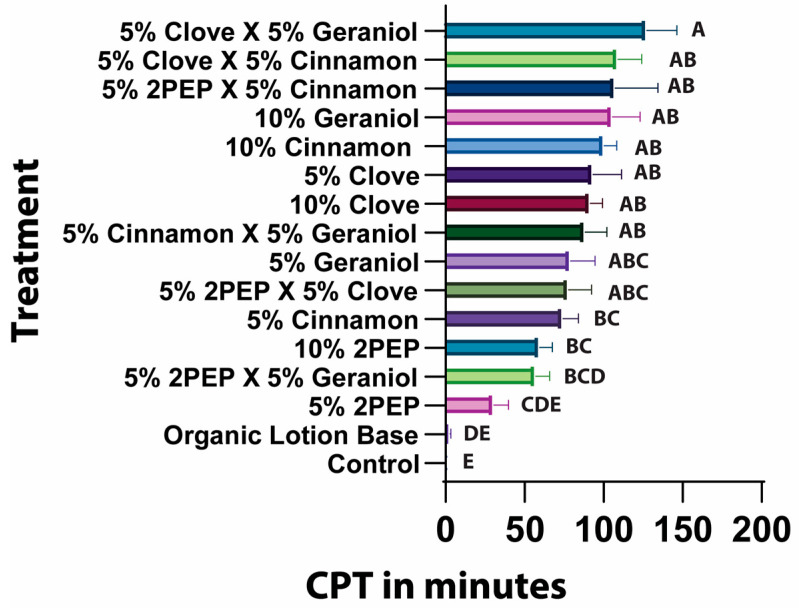
CPTs of different combinations of plant-based oils. CPTs were measured using an arm-in-cage assay. A one-way ANOVA followed by Dunnett’s multiple comparison test were used to calculate the statistical significance (*p* < 0.05) between the mean and standard deviation of each group. Experimental groups that share the same letter indicate no statistical difference, while groups that do not share the same letter indicate a statistically significant difference (*p* < 0.05) between the groups.

Next, we tested the repellent efficacy of different mixtures and concentrations of clove and cinnamon oil, geraniol, and 2PEP. All combinations we tested produced significantly greater CPTs than the organic lotion base control (Figure 3). Clove oil combined with geraniol had the longest CPT out of all the combinations. This combination had a significantly longer CPT than 5% geraniol but not 5% clove oil alone. Furthermore, 5% 2PEP combined with 5% cinnamon significantly increased the CPT when compared with 5% 2PEP alone. These combinations were not significantly different than 5% clove oil or 5% cinnamon oil.

### 3.4. Mosquito Repellency Assessment of Major Clove and Cinnamon Oil Constituents

**Figure 4:**
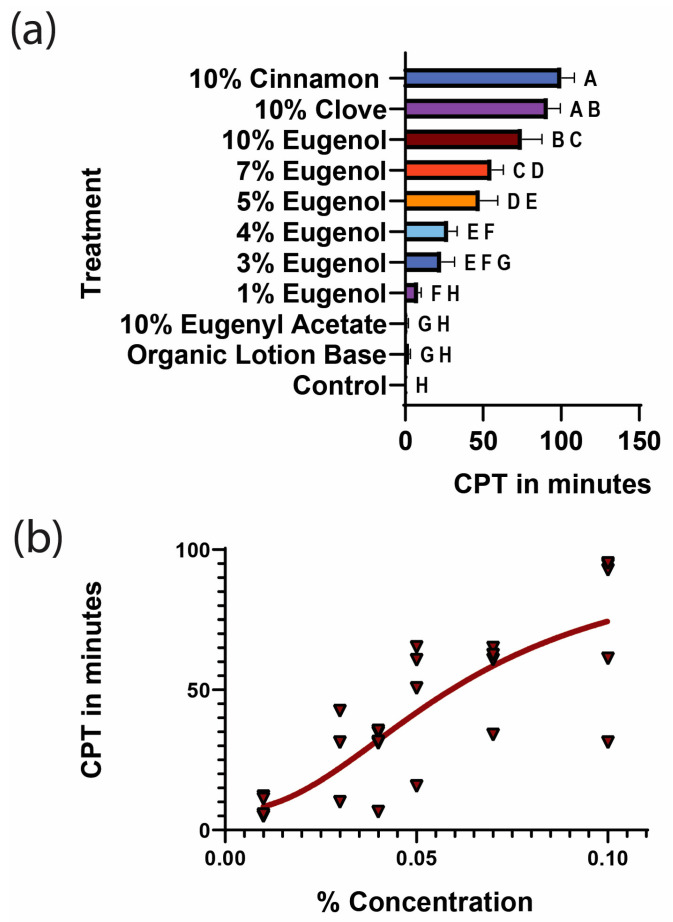
Repellent efficacy of the compounds eugenol and eugenyl acetate. CPT was measured using the arm-in-cage assay. (**a**) Eugenyl acetate and a dilution series of eugenol were tested. A one-way ANOVA followed by Dunnett’s multiple comparison test were used to calculate the statistical significance (*p* < 0.05) between the mean and standard deviation of each group. Experimental groups that share the same letter indicate no statistical difference, while groups that do not share the same letter indicate a statistically significant difference (*p* < 0.05) between the groups. (**b**) Eugenol curve model fit. Individual data points (red triangles) are overlaid with a four-parameter sigmoidal curve model fitted to the data for a dilution series of eugenol (red line). EC50 = 6.2. Hillslope = 2.2. The goodness of fit was determined based on corresponding R^2^ = 0.65.

The major components in clove oil are eugenol, an oxygenated monoterpene, and eugenyl acetate, an ester (see Supplemental File S1). We tested a 10% eugenyl acetate emulsion and eugenol at various concentrations in the arm-in-cage assay (see Figure 4). Eugenyl acetate did not provide a CPT significantly different from the organic lotion base control. A 10% eugenol formulation provided a CPT statistically similar to clove oil but not cinnamon oil.

## 4. Discussion

Effective mosquito repellents are heavily relied on for the protection of consumers from mosquito bites and mosquito-borne diseases [44]. There is growing interest in plant-based “natural” repellents as opposed to “chemical”, synthetic products with active ingredients like DEET, picaridin, or IR3535. While consumer interest is growing in plant-based oils as mosquito repellents, the current gold standard for protection from mosquito bites continues to be DEET. The repellent efficacy of novel products that rely on the repellency of plant-based essential oils or other ingredients should be scientifically evaluated to inform their labels. An aim of this study was to provide scientific data to support the development and formulation of novel mosquito repellents from “natural” ingredients.

Our first goal was to identify lead compounds that contribute to the repellency of these plant-based oils [36]. The chemical composition of different batches can differ significantly, even when these oils are isolated from the same plants [45,46]. The variables responsible for this diversity are the method of distillation, the plant parts used for extraction, plant strains, season and climate during the growth season, and the geographic region that the plants are sourced from [47,48,49,50,51,52]. For example, the cinnamon oil we sourced from Sigma Aldrich (St. Louis, MO, USA.) (Table 1) contained a relatively high concentration of eugenol, indicating it is likely derived from the leaves and not the bark, which typically contains a high amount of cinnamaldehyde [53,54]. On the other hand, the clove oil we also sourced from Sigma Aldrich contained characteristic compounds such as eugenol and eugenyl acetate in similar quantities found in clove oil from other sources and lacked the expected abundance of caryophyllene [55,56]. We hypothesize that variations in specific compound concentrations can change the efficacy of any given essential oil as a mosquito repellent. This might explain the large number of conflicting reports on the effectiveness of citronella essential oil as a mosquito repellent [14,57,58].

When we re-analyzed the composition of plant-based essential oils (see Supplemental File S1), we found that most of the analyzed oils were complex mixtures of various compounds. Interestingly, the oils that provided the longest protection from mosquito bites were the least complex amongst the oils (see Figure 1). Notably, clove oil and cinnamon oil only consist of three compounds while geraniol and 2-PEP are pure compounds. Our results suggest that the repellency of these plant-derived essential oils is due to active components that are in low abundance.

Plant-based essential oils are the most expensive ingredient of essential oil-based mosquito repellents [59,60]. Commercial repellents therefore often contain relatively low concentrations of these oils, typically between 1 and 10%. In addition, some essential oils can be skin irritants at high concentrations [61]. There is very little scientific literature that has systematically investigated the impacts of low concentration of 25b essential oils and their repellent efficacy. One study from 1999 conducted by Barnard investigated the repellent efficacy of oils from the EPA 25(B) list in a 5–100% concentration range using ethanol as a carrier [35]. He reported that clove oil at concentrations of 5 and 10% provided no protection from mosquito bites and that the higher the concentration, the longer the complete protection, with 25% protecting for 90 min and 100% protecting for 225 min.

We hypothesize that the carrier used with plant-based oils is critical for their efficacy as mosquito repellents. We found in the current and in other studies that clove oil in an organic lotion base can confer protection from mosquito bites for over an hour at 10% concentrations. The results from our dilution series experiments (Figure 2) in which we used an organic lotion base as a carrier support this hypothesis because the CPTs were significantly longer than those found by Barnard and coworkers when using ethanol as a carrier [35].

Our findings suggest that the concentrations of individual plant-based oils are critical for the length of the CPT. We observed sigmoid curves when we plotted the CPTs of clove oil, cinnamon oil, 2-PEP, and geraniol against their concentrations in the repellent mixtures. We found that the tested oils had a sharp increase in CPT once a minimum effective dose (MED) was reached. For example, when testing dilutions of geraniol, we observed a 16-fold increase in CPT between 4% and 5% concentrations. We also found some evidence to suggest that these oils have an optimal concentration that confers the longest protection from mosquito bites. This was seen with clove oil, where the 10% concentration protected for the same length of time as the 5% concentration. This suggests that 5% clove oil in an organic lotion base is the optimal concentration to confer the longest CPT. The research conducted by Deng and coworkers in 2023 supports our findings. When they tested clove oil at concentrations higher than 10%, they found no increase in CPT [62]. We predict that once this concentration is reached, higher concentrations will not confer longer CPTs. However, it is important to note that the use of other carriers will likely result in different optimal concentrations.

Many commercially available essential oil-based mosquito repellents contain mixtures of two or more different essential oils. While the rationale for mixing different essential oils in these repellents is not explained on any label we analyzed, it is likely that some sort of positive combination effect is expected. There is abundant literature supporting additive and synergistic effects after combining different plant-based oils for a variety of applications including insect repellency [63,64,65,66]. Our experiments using binary mixtures of essential oils/compounds were designed as a preliminary screen for potential additive and antagonistic effects. Surprisingly, we found neither effect (Figure 3). Our combinations of oils did not provide any significant increases in CPT when compared with their constituent individual oils at a 5% concentration.

Lastly, we tested the hypothesis that the repellent activity of clove and cinnamon oil is due to a specific constituent shared by both oils. The two major constituents of clove and cinnamon oil are eugenol and eugenyl acetate. Eugenol is a phenolic oxygenated monoterpene with well-known antibacterial, acaricidal and insecticidal activities [67,68,69]. Interestingly, when testing eugenol in a dilution series, we did not see a sharp increase in CPT between specific consecutive concentrations as we did in clove, cinnamon, 2PEP, and geraniol. Instead, we saw a very linear increase in CPT as eugenol concentration increased (Figure 4). We also showed that 10% cinnamon oil conferred significantly longer CPTs than 10% eugenol. To expand on this finding, we tested the hypothesis that the compound eugenyl acetate has repellent properties. However, 10% eugenyl acetate did not show any bioactivity in our tests. We predict that this compound may work in synergy with eugenol to confer the CPT of cinnamon and clove oil. More focused studies are necessary to answer this question. The results of our study suggest that the repellent activity of specific essential oils is caused by only a single or a small number of active compounds in these oils. When mixed, different compounds did not show additive or synergistic effects when tested as repellents. Therefore, mixing different essential oils might not be a useful strategy when creating new formulations of plant-based mosquito repellents. In order to better understand these findings, we suggest a controlled study of individual active ingredients, addressing their evaporation profiles in a repellent mixture as well as their skin absorption and release patterns when applied to human skin. Determining the optimal concentration for individual essential oils in specific carriers will be valuable for the formulation of novel essential oil-based repellents.

## 5. Conclusions

Our study highlights the importance of optimizing concentrations and combinations of plant-based oils and their carriers when formulating novel mosquito repellents that are based on active ingredients from the EPA 25(b) list. The fact that the chemical composition of plant EOs can vary significantly depending on plant parts, season, geographical location, and method of extraction must be considered. Our results suggest that the repellent efficacy of plant-based oils is more likely due to a small number of active compounds rather than to additive or synergistic effects in complex mixtures.

## Figures and Tables

**Table 1:** Oils and compounds used in this study.

Common Name	Millipore Sigma Number	CAS Number
Cinnamon oil (Ceylon Type) (*Cinnamomum verum* (Presl, 1823))	W229202	8015-91-6
Clove oil (*Syzygium aromaticum* (L.) Merr, L.M. Perry, 1939)	C8392	8000-34-8
Eugenol	W246719	97530
Eugenyl Acetate (Chavibetol acetate)	W246905	93-28-7
Geraniol	163333	106-24-1
2-Phenylethyl propionate (2PEP)	W286702	122-70-3

## Data Availability

All data generated or analyzed during this study are included in this publication and its Supplemental Files.

## Supplementary Materials

The following supporting information can be downloaded at https://www.mdpi.com/article/10.3390/insects16010051/s1, File S1: GCMS profiling data; File S2: Arm-in-cage raw data.
